# Comparison of dietary intake measured by a web-based FFQ and repeated 24-hour dietary recalls: the Hordaland Health Study

**DOI:** 10.1017/jns.2022.97

**Published:** 2022-11-04

**Authors:** Zoya Sabir, Hanne Rosendahl-Riise, Jutta Dierkes, Helene Dahl, Anette Hjartåker

**Affiliations:** 1 Centre for Nutrition, Mohn Nutrition Research Laboratory, Department of Clinical Medicine, University of Bergen, Bergen, Norway; 2 Division of Nutritional Epidemiology, Department of Nutrition, University of Oslo, Oslo, Norway

**Keywords:** Dietary assessment, FFQ, The Hordaland Health Study, Validation, WebFFQ, 24-HDR, 24-hour dietary recall interviews, BMI, body mass index, CI, confidence interval, DLW, doubly labelled water, FFQ, food frequency questionnaire, HUSK3, The Hordaland Health Study, KBS, KostBeregningsSystemet, kJ, kilojoule, LOA, limits of agreement, p25–p75, 25th percentile–75th percentile, sd, standard deviation, WebFFQ, web-based food frequency questionnaire

## Abstract

All dietary assessment methods inevitably introduce measurement errors, which should ideally be considered during data analysis and interpretation. Methodological studies should be conducted to address how well a given assessment method captures dietary intake and to highlight the extent and direction of the measurement error. Within a subgroup of the Hordaland Health Study (HUSK3), we examined the relative validity of a web-based food frequency questionnaire (WebFFQ) by comparing its estimates of mean daily intake of nutrients and foods with estimated mean daily intakes from repeated administrations of 24-hour dietary recall interviews (24-HDRs). Men and women born between 1950 and 1951 were recruited from HUSK3. The participants (*n =* 67) completed a WebFFQ and three non-consecutive 24-HDRs over the course of a year. Relative validity was assessed using Spearman's rank correlation, crosstab analysis and Bland–Altman plots. Linear regression models were used to compute the calibration coefficients. The estimated correlation coefficients were acceptable or strong for all nutrients and foods except iodine (*r_s_* = 0⋅19). The highest correlation coefficient was found for juice (*r_s_* = 0⋅71), whereas the lowest correlation coefficient was found for iodine (*r_s_* = 0⋅19). Cross-classification by quartiles categorised more than 72 % of the participants into the same or adjacent quartiles using the two methods. Few data points fell outside the limits of agreement in the Bland–Altman plots. Calibration coefficients ranged from 0⋅10 (wholegrain) to 0⋅81 (alcohol). Our findings suggest that the WebFFQ has reasonable ranking abilities for all the included nutrients and foods, except for iodine.

## Introduction

Adequate exposure assessment is essential in all epidemiological studies, although it may be particularly challenging in nutritional epidemiology^(1)^. Dietary intake represents a complex and multidimensional exposure, largely conditioned by culture and geographic location. Being a dynamic exposure, dietary intake is prone to variation across days, weeks, seasons and the lifecycle^(2)^. However, the global recognition of diet as a major modifiable risk factor for various non-communicable diseases implies that measuring and targeting diet remains essential^(3)^. In the absence of objective biomarkers for overall diet, nutrition researchers typically administer methods to obtain self-reported dietary intake^(4)^, inevitably introducing measurement errors which should be considered during data analysis and interpretation^(1,5)^. Hence, assessing the validity of such dietary assessment tools is crucial to ensure that diet-disease associations are reported as precisely as possible^(6)^. Owing to the unfeasibility of accurately measuring the self-selected diet of individuals over longer time periods, studies often assess relative validity by comparing the test method with an alternative method for dietary intake assessment that has its own set of limitations^(7,8)^.

The food frequency questionnaire (FFQ) is designed to retrospectively measure habitual long-term dietary intake and is the most frequently applied dietary assessment tool in large-scale epidemiological studies^(9,10)^. Despite the feasibility and cost-effectiveness of FFQs, various biomarker studies have raised concerns regarding the validity of FFQs and their ability to detect moderate diet-disease relationships^(11)^. To obtain adequate estimates for ranking individuals according to intake levels, de Boer *et al.* recommended administering at least two non-consecutive 24-hour dietary recall interviews (24-HDRs) combined with a food propensity questionnaire^(12)^. Although the 24-HDR does not produce unbiased data, its data have been reported to be less prone to bias than the FFQ in several populations. Hence, the 24-HDR may be used as a reference method in relative validation studies of the FFQ^(13,14)^.

The Hordaland Health Study (HUSK3) is a population-based survey designed to collect health data that may contribute to disease prevention. HUSK3 included the assessment of dietary intake, completed by the administration of an extensive web- and image-based FFQ (WebFFQ), as well as 24-HDRs. It is recommended that estimates of validity are obtained from subjects who are representative of the main study target population^(6,15,16)^. Hence, in a subgroup of the HUSK3 study population, we aimed to assess the relative validity of the WebFFQ in estimating energy, nutrient and food intake by comparing its measures with those derived from repeated, non-consecutive 24-HDRs.

## Methods

### Study design and subjects

From 1992 to 1993, all residents of Hordaland County born in 1925–27 and 1950–52 were invited to participate in the Hordaland Homocysteine Study. From 1997 to 1999, participants belonging to the 1925–27 and 1950–51 birth cohorts were reinvited to participate in HUSK2. Dietary data collection was introduced in HUSK2 through a 169-item semi-quantitative paper-based FFQ (PB-FFQ), developed at the Department of Nutrition, University of Oslo, Norway.

In 2018, the follow-up health survey, HUSK3, was initiated as a joint project between the University of Bergen, Helse Bergen HF, and the Public Dental Health Service Competence Centre for Western Norway (TkVestland). In HUSK3, men and women born in 1950–51, who had previously taken part in HUSK2 were recruited. HUSK3 is a population-based observational study consisting of 2232 men and women. Participants were enrolled and health examinations were conducted from 2018 to 2020. HUSK3 included measurements of body weight, height, blood pressure, body composition, handgrip strength, as well as echocardiography, blood tests and assessment of dietary intake. Dietary intake information was collected from all HUSK3 participants through a single in-person 24-HDR on the HUSK3 survey day, and all participants received information on how to access the WebFFQ to be completed at home, following the HUSK3 survey day.

Within a subsample of HUSK3, we conducted a study with the aim of addressing methodological challenges in future analyses using dietary intake data collected in HUSK3 to assess diet-health associations. In the present study, we compare measures of nutrient and food intake from the HUSK3 WebFFQ with measures of nutrient and food intake from three repeated 24-HDRs. In the present comparison study, 175 men and women who underwent the HUSK3 health survey between January and March 2020 granted their informed written consent to participate. In addition to the in-person 24-HDR and WebFFQ administered to all HUSK3 participants, the participants in the comparison study agreed to provide additional dietary information through telephonic 24-HDRs.

On the HUSK3 survey day, the first 24-HDR was conducted, and participants were given a link to access the WebFFQ, which they were requested to complete at home. Hence, the WebFFQ was administered after the first 24-HDR, but prior to conducting the second and third 24-HDR for the comparison study. However, participants were recruited sequentially as they came to attend the HUSK3 survey at the Research Unit for Health Surveys (RUHS) – and therefore filled out the WebFFQ at different time points. Within the comparison study, the in-person 24-HDR conducted on the survey day was regarded as the first administration. The second administration of 24-HDR was completed via telephone by 121 participants included in the comparison study, while the third 24-HDR was completed via telephone by 68 of the included participants. The study was conducted in accordance with the Declaration of Helsinki, and dietary data collection was approved by the Regional Committees for Medical and Health Research Ethics West (REK 2017/294).

### Data collection methods

#### The Web-based FFQ

The WebFFQ used in HUSK3 was developed by the Department of Nutrition, Institute of Basic Medical Sciences, University of Oslo. The validity of the WebFFQ has previously been assessed by Medin *et al.* in adults aged 18–70 years^(3)^. The self-administered WebFFQ consists of 279 items intended to measure the habitual intake of the following foods: bread, sandwich spreads, cereals, yoghurt, non-alcoholic and alcoholic beverages, hot dishes containing meat and fish/seafood, potatoes, rice, pasta, vegetables, sauces and condiments, fruits and berries, nuts and seeds, desserts, cakes and pastries, chocolate and sweets, types of cooking fat, and fat used on bread. Furthermore, the WebFFQ enquired about dietary supplement use. Finally, an open field allowed participants to report the consumption of foods or supplements that were not already included in the WebFFQ.

Frequency of consumption was inquired for all food items, ranging from *never* to *multiple times daily*, with the number of frequency response options and intervals varying depending on the food item in question. Portion sizes were also estimated for all food items, although in different ways. For some food items (e.g. bread, sausages, hamburgers, spring rolls, sushi, fish cakes, taco shells), the WebFFQ inquired about ‘*number of slices/pieces*’ consumed of an assumed standard portion size (e.g. number of sausages). For food items typically difficult to estimate in units (e.g. breakfast cereals, pizza, lasagna, stews, wok, nuts), portion sizes were estimated by using pictures of four different portion size alternatives. Each picture corresponded to a predefined quantity (grams) of food. The standard portion sizes applied in the WebFFQ are based on values from ‘Weights, measures and portion sizes for foods’^(17)^.

Information on the intake of food items, energy and nutrients was generated using KostBeregningsSystemet (KBS, version 7.4, database AE18), a food and nutrient composition database and calculation system developed at the Department of Nutrition, Institute of Basic Medical Sciences, University of Oslo. The AE18 database is based on the official Norwegian Food Composition Table version 2018, with added items, including approximately 3400 food items^(18)^. When the consumption frequency was reported as a range (e.g. 1–2 times per week), the mean frequency was applied. Missing frequencies and portion estimates were avoided by built-in error checks, meaning that participants could not proceed to the next question unless all boxes were ticked off for each question. To estimate the daily intake of food, energy and nutrients, the recorded frequency was multiplied by the corresponding portion size. Fat used for bread and cooking, as well as supplements, was considered in the nutrient calculations. The mixed dishes were split into separate ingredients, which allowed each ingredient to be categorised into the most appropriate food group (e.g. pizza was split into cheese, meat, vegetables, flour). The splitting of foods did not affect the calculation of nutrients. The foods and nutrients included for the food/food group analysis were selected prior to data analysis. The included foods represent the main food groups enquired about in the WebFFQ, whereas nutrients were selected due to their presumed relevance to future studies of diet-disease associations^(19,20,21)^.

#### The 24-hour dietary recall interviews

The 24-HDR was chosen as the reference method to assess the relative validity of the WebFFQ. The first 24-HDR was conducted in person as an integrated part of the HUSK3 health survey, using a paper-based approach. The in-person 24-HDR applied a three-part approach, approximately similar to that of the telephonic 24-HDRs described below. However, in contrast to the telephonic 24-HDRs, it did not apply a picture booklet to facilitate portion size estimation. Rather, the in-person 24-HDR required participants to report amounts in household measurements (e.g. cups, tablespoons), natural units (e.g. number of slices/pieces) and standard units of measurement (grams, decilitres). The second and third 24-HDR rounds were conducted via telephone using an integrated 24-hour multi-pass recall module in KBS, which was connected to its nutrition composition database (KBS, version 7.4, database AE18). The method has previously been described by Myhre *et al.*^(22)^ and involves three steps. First, the participant provides a free description of the meals that were consumed the day prior to the interview. Second, the interviewer restates all reported items while adding follow-up questions regarding portion sizes, potential forgotten items and meals. Finally, the interviewer probes for frequently overlooked items such as snacks and supplements.

To facilitate portion estimation, the participants received a booklet containing pictures of different portion sizes prior to the telephonic 24-HDRs. The picture booklet used for the telephonic 24-HDRs was originally developed for use in Norkost3, which is the third national dietary survey in Norwegian adults conducted between 2010 and 2011^(23)^. The booklet included pictures of plates, bowls, glasses and cups of different volumes. This was followed by pictures of bread rolls, baguettes and nine illustrations of bread slices of oval and squared shapes. Each illustration of bread referred to two to three different thickness alternatives. Finally, the booklet included pictures of thirty-three foods/dishes, with four different portion sizes ranging from small (A) to large (D) for each food. Each portion size alternative in the picture booklet corresponds to a weight in grams. The selection of pictures was based on experiences from a previously conducted Norwegian survey, Ungkost 2000^(24)^, as well as a booklet used in the European validation project, EFCOVAL^(25)^. The illustrations of bread have been obtained from a picture booklet used in The Norwegian Women and Cancer Study (NOWAC) at The University of Tromsø^(26)^. To facilitate the reporting of bread type, the picture booklet included the Bread Scale symbol for the level of wholegrain content, developed by the Federation of Norwegian Bakers and Confectioners.

To standardise the process, all 24-HDRs included in the comparison study were performed by the same clinical dietitian (ZS). All recall interviews used in this study were collected between January 2020 and January 2021 and were spread out evenly to account for day-to-day and seasonal variations. Fifty-four (27 %) of the recall interviews included in this study reflected dietary intake on weekend days. To avoid conscious or subconscious alterations in dietary intake according to social desirability, the interviewees were not informed of the days they would be interviewed on in advance. KBS software and its food composition database were used to estimate food, energy and nutrient intake from the 24-HDRS. Mixed dishes were split into separate ingredients, allowing each ingredient to be categorised into the most appropriate food group.

### Statistical analyses

Background characteristics are presented as medians with percentiles (p25–p75) for non-normally distributed variables. Categorical variables are presented as proportions (%). Due to the predominantly skewed distributions of nutrient and food intake data, these are presented as medians with the corresponding 25th and 75th percentiles. Normality was assessed using the Kolmogorov–Smirnov test and by examination of the associated Q-Q plots and histograms. Dietary supplements were included in the nutrient intake estimates from the WebFFQ and the 24-HDRs. Adjustment for total energy intake (kilojoules) was carried out using the simple nutrient density model as described by Willet *et al.*^(27)^.

The Wilcoxon signed-rank test was applied to assess the differences between the estimated nutrient and food intake from the WebFFQ and repeated 24-HDRs. The relative validity of the WebFFQ was assessed by computing Spearman's rank-order correlation coefficients, reflecting the degree to which the WebFFQ and the 24-HDRs ranked participants equally in terms of nutrient and food intake. In accordance with previous dietary validation studies^(8,28)^, the following Spearman's rho cut-offs were applied to categorise the strength of the correlations: poor <0⋅20, acceptable 0⋅20–0⋅49 and strong ≥0⋅50.

Cross-classification tables were created to evaluate the extent to which the WebFFQ classified participants into the same quartiles of dietary intake as the repeated 24-HDRs. Due to non-reporting in the 24-HDRs, proper cross-classification tables could not be computed for alcohol and some foods. Agreement between methods is represented by the proportion of participants classified in the same/adjacent or opposite quartile by the WebFFQ compared with the 24-HDRs.

The agreement between the WebFFQ and 24-HDRs was also assessed using Bland–Altman plots, visualising the difference in intake between the two methods plotted against the mean intake of the two methods. Limits of agreement (LOAs) were calculated as the mean difference ± 1⋅96 sd, providing an interval that includes the difference between single measurements on the same participant by the two methods with 95 % probability. Calibration coefficients (λ) were derived using linear regression of the 24-HDR data (dependent variable) on the FFQ data (independent variable).

All reported *P*-values are two-sided, and a statistical significance level of *P* < 0⋅05 was applied. All statistical analyses were performed using Statistical Package for Social Sciences version 25, IBM Corporation (IBM Corp. Released 2017. IBM SPSS Statistics for Windows, version 25.0. Armonk, NY: IBM Corp).

## Results

Among the sixty-eight participants who completed three repetitions of the 24-HDR, one participant was excluded due to a lack of WebFFQ data. The background characteristics of the participants included in the analysis are presented in Table 1. Among the sixty-seven participants who completed the WebFFQ and three repetitions of the 24-HDRs, 60 % were female, the median body mass index (BMI) was 24⋅9 kg/m^2^ and the median waist circumference was 104⋅5 cm in males and 86⋅0 cm in females. 5 % of the participants were current smokers. With regard to the mentioned characteristics, the participants completing the comparison substudy were representative of the main HUSK3 study population.

**Table 1.** Characteristics of the participants completing the comparison study and the total Hordaland Health Study 3 cohort

Characteristic	Subjects completing 3 × 24-HDR and WebFFQ (*n* 67)	Subjects in the HUSK3 cohort (*n* 2232)
Female (%)	60	54
Body weight, measured (kg)^a^	71⋅9 (65⋅4–89⋅9)	76⋅3 (66⋅9–86⋅7)^b^
Height, measured (cm)^a^	169⋅0 (165⋅0–177⋅0)	170⋅0 (164⋅0–178⋅0)^b^
BMI, measured (kg/m^2^)^a^	24⋅9 (23⋅4–28⋅5)	26⋅0 (23⋅7–28⋅6)^b^
Waist, measured (cm)^a^		
Men	104⋅5 (98⋅8–111⋅5)	100⋅0 (94⋅0–107⋅0)^b^
Women	86⋅0 (79⋅0–92⋅5)	90⋅0 (82⋅0–99⋅0)^b^
Current smoker (%)	5	8

24-HDR, 24-hour dietary recall; WebFFQ, web-based food frequency questionnaire; BMI, body mass index.

a Median (25th percentile–75th percentile).

b Weight data available for 2182 participants. Height data available for 2178 participants. BMI data available for 2173 participants. Waist data available for 984 men and 1192 women.

Table 2 presents the absolute and energy-adjusted intakes of energy-providing nutrients including dietary fibre and alcohol, selected micronutrients and various food groups from the WebFFQ and the mean of three 24-HR dietary. Estimated absolute intakes from the WebFFQ were significantly higher for twenty-six out of thirty nutrients and foods. The largest overestimations in absolute intake by the WebFFQ were observed for ‘vegetables’, iodine and calcium. The only food significantly underestimated by the WebFFQ was ‘cheese’. No significant differences in absolute intakes between the WebFFQ and the 24-HDRs were evident for added sugar, alcohol and ‘eggs’. Energy adjustment of intake led to a prominent decline in the number of overestimated nutrients and foods by the WebFFQ, with only eleven nutrients and foods being significantly overestimated, seventeen being similar, and two being significantly underestimated.

**Table 2.** Absolute and energy-adjusted intakes of nutrients and foods, and Spearman's correlation coefficients between estimated absolute (*r_s_*) and energy-adjusted (*r_a_*) intakes from the WebFFQ and the mean of three 24-hour dietary recalls in a comparison study among Norwegian adults participating in the Hordaland Health Study 3

	Absolute intake (unit/day)			Energy-adjusted intake (unit/1000 kcal)		
	WebFFQ	3 × 24-HDR^†^		WebFFQ	3 × 24-HDR^†^	
Nutrients and food groups	Median (p25–p75)	Median (p25–p75)	Spearman's *r_s_*	Median (p25–p75)	Median (p25–p75)	Spearman's *r_a_*
Energy^a^ (kJ/kcal)	11 288 (7855–15 048)	7629 (6638–9155)*	0⋅52**	2698 (1877–3597)	1823 (1587–2188)*	0⋅52**
Protein (g)	114 (87–148)	79 (67–94)*	0⋅50**	44 (40–49)	44 (40–48)	0⋅36**
Fat (g)	104 (74–150)	72 (58–93)*	0⋅50**	40 (36–47)	40 (36–44)	0⋅39**
Saturated fat (g)	33 (23–52)	26 (21–35)*	0⋅46**	13 (11–15)	15 (12–16)	0⋅31**
MUFA (g)	40 (27–55)	27 (21–35)*	0⋅51**	15 (13–18)	14 (13–16)*	0⋅39**
PUFA (g)	21 (15–29)	13 (11–17)*	0⋅43**	8 (6–9)	7 (6–8)*	0⋅36**
Omega-3 fatty acids (g)	5 (3–7)	3 (2–5)*	0⋅57**	2 (1–3)	2 (1–3)*	0⋅63**
Carbohydrates (g)	242 (197–332)	191 (154–231)*	0⋅47**	102 (92–109)	103 (93–112)	0⋅33**
Added sugar (g)	21 (15–36)	22 (13–34)	0⋅51**	9 (6–13)	12 (7–16)*	0⋅52**
Dietary fibre (g)	37 (29–53)	21 (17–27)*	0⋅45**	15 (13–17)	12 (10–14)*	0⋅48**
Wholegrain (g)	90 (54–123)	53 (35–81)*	0⋅27*	37 (24–54)	29 (22–41)*	0⋅23**
Alcohol (g)	4 (1–7)	0 (0–11)	0⋅53**	1 (1–3)	0 (0–6)	0⋅49**
Vitamin D (μg)	21 (10–30)	15 (6–25)*	0⋅69**	6 (4–11)	8 (4–13)	0⋅72**
Calcium (mg)	979 (793–1370)	821 (583–1090)*	0⋅44**	391 (315–533)	428 (334–574)	0⋅61**
Iodine (μg)	324 (229–454)	158 (103–256)*	0⋅19	129 (97–169)	80 (59–123)*	0⋅15
Bread (g)	172 (94–257)	125 (85–191)*	0⋅50**	69 (46–99)	72 (53–94)	0⋅38**
Cereals (g)	76 (40–157)	50 (10–127)*	0⋅41**	34 (16–55)	23 (9–63)	0⋅37**
Potatoes (g)	84 (53–119)	60 (33–97)*	0⋅38**	31 (17–48)	28 (15–54)	0⋅33**
Vegetables (g)	340 (278–613)	139 (93–207)*	0⋅32**	161 (103–222)	71 (58–101)*	0⋅41**
Fruits and berries (g)	358 (208–572)	215 (122–375)*	0⋅68**	149 (103–192)	119 (65–190)	0⋅63**
Juice (g)	59 (6–159)	33 (0–140)*	0⋅71**	20 (2–71)	27 (0–79)	0⋅70**
Meat, blood, offal (g)	123 (79–168)	82 (53–138)*	0⋅32**	44 (34–60)	50 (27–72)	0⋅44**
Red meat (g)	91 (55–126)	66 (37–103)*	0⋅47**	33 (21–47)	35 (23–55)	0⋅55**
Fish and shellfish (g)	133 (77–184)	73 (17–117)*	0⋅34**	47 (34–70)	36 (10–70)	0⋅31*
Lean fish (g)	32 (20–55)	0 (0–33)*	0⋅26*	13 (8–26)	0 (0–21)*	0⋅25*
Fatty fish (g)	30 (17–46)	0 (0–29)*	0⋅31*	11 (7–16)	0 (0–23)*	0⋅31**
Eggs (g)	24 (13–44)	19 (0–37)	0⋅32**	9 (5–15)	12 (0–21)	0⋅31**
Milk, yoghurt (g)	227 (124–510)	200 (92–305)*	0⋅57**	94 (56–185)	98 (65–167)	0⋅60**
Cheese (g)	20 (10–36)	29 (17–48)*	0⋅56**	8 (4–13)	19 (9–24)*	0⋅51**
Butter, margarine, oil (g)	34 (15–71)	18 (6–31)*	0⋅61**	11 (7–23)	8 (4–15)*	0⋅61**

kJ, kilojoule; kcal, kilocalories; FFQ, food frequency questionnaire; 24-HDR, 24-hour dietary recall; p25, 25th percentile; p75, 75th percentile; MUFA, monounsaturated fatty acids; PUFA, polyunsaturated fatty acids; *r_s_*, Spearman's correlation coefficient; *r_a_*, energy-adjusted Spearman's correlation coefficient.

a kJ for absolute intake. kcal for energy-adjusted intake.

† Intake from the WebFFQ compared with intake from the repeated 24-HDRs using the Wilcoxon signed-rank test.

* Statistically significant difference from estimated WebFFQ intake | Statistically significant Spearman's rank correlation. The significance level was set at *P* < 0⋅05.

** Statistically significant Spearman's rank correlation. The significance level was set at *P* < 0⋅01.

Spearman's correlation coefficient between estimated intakes from the WebFFQ and 24-HDRs (Table 2) ranged from 0⋅19 (iodine) to 0⋅69 (vitamin D) for nutrients and from 0⋅31 (fatty fish) to 0⋅71 (juice) for foods. Correlations were statistically significant for all nutrients and foods, except iodine. All correlations remained statistically significant after energy adjustment. Improvements in correlation coefficients were observed for nine out of thirty nutrients and food when energy-adjusted, with the most prominent improvement for calcium, ‘meat, blood, offal’ and ‘vegetables’. However, energy adjustment led to the largest decline in the correlation coefficients for saturated fat, protein and carbohydrates.

The stability of quartile membership was assessed by cross-classification of intake estimates from the WebFFQ and 24-HDRs, as shown in Table 3. For most nutrients and foods, extreme misclassification did not exceed 5 %. The highest degree of similar classification was observed for ‘fruits and berries’ and vitamin D, while the lowest degree of similar classification was observed for iodine, ‘red meat’ and ‘meat, blood, offal’.

**Table 3.** Agreement of quartile membership, mean differences with limits of agreement between estimated absolute daily intakes of energy, nutrients and foods from the 24-HDRs and WebFFQ, and the calibration coefficient in the Hordaland Health Study 3

Nutrients and food groups	Cross-classifications					Calibration	
	Same/adjacent quartile	Extreme opposite quartile	Mean difference (sd)^†^	95 % LOA^‡^		λ (95 % CI)	λ^a^ (95 % CI)
Energy (kJ)	78 %	1 %	3763 (4247)	−4560	12 086	0⋅20 (0⋅10–0⋅31)	0⋅20 (0⋅10–0⋅31)
Protein (g)	85 %	3 %	40 (44)	−45	126	0⋅17 (0⋅07–0⋅27)	0⋅41 (0⋅15–0⋅67)
Fat (g)	84 %	3 %	42 (55)	−66	149	0⋅16 (0⋅07–0⋅26)	0⋅36 (0⋅17–0⋅55)
Saturated fat (g)	81 %	1 %	12 (23)	−33	57	0⋅13 (0⋅02–0⋅23)	0⋅31 (0⋅08–0⋅55)
MUFA (g)	87 %	3 %	16 (20)	−22	55	0⋅18 (0⋅09–0⋅27)	0⋅29 (0⋅11–0⋅48)
PUFA (g)	79 %	6 %	10 (13)	−15	35	0⋅18 (0⋅09–0⋅26)	0⋅28 (0⋅16–0⋅39)
Omega-3 fatty acids (g)	87 %	3 %	2 (3)	−3	8	0⋅28 (0⋅15–0⋅40)	0⋅61 (0⋅40–0⋅81)
Carbohydrates (g)	78 %	1 %	85 (111)	−133	302	0⋅23 (0⋅11–0⋅34)	0⋅41 (0⋅20–0⋅63)
Added sugar (g)	85 %	3 %	3 (18)	−33	39	0⋅38 (0⋅17–0⋅60)	0⋅64 (0⋅37–0⋅90)
Dietary fibre (g)	85 %	1 %	19 (17)	−13	52	0⋅21 (0⋅11–0⋅30)	0⋅56 (0⋅36–0⋅75)
Wholegrain (g)	75 %	4 %	48 (84)	−117	214	0⋅10 (−0⋅01 to 0⋅20)	0⋅23 (0⋅03–0⋅42)
Alcohol (g)	n/a	n/a	-2 (13)	−27	23	0⋅81 (0⋅37–1⋅24)	0⋅86 (0⋅29–1⋅43)
Vitamin D (μg)	88 %	1 %	5 (11)	−17	27	0⋅55 (0⋅42–0⋅67)	0⋅78 (0⋅62–0⋅93)
Calcium (mg)	82 %	4 %	322 (592)	−839	1483	0⋅22 (0⋅08–0⋅36)	0⋅45 (0⋅24–0⋅66)
Iodine (μg)	72 %	7 %	190 (215)	−231	610	0⋅15 (0⋅01–0⋅29)	0⋅29 (0⋅06–0⋅51)
Bread (g)	79 %	3 %	75 (186)	−290	441	0⋅10 (0⋅00–0⋅20)	0⋅30 (0⋅12–0⋅49)
Cereals (g)	76 %	4 %	42 (103)	−161	244	0⋅23 (0⋅07–0⋅38)	0⋅42 (0⋅15–0⋅68)
Potatoes (g)	70 %	3 %	34 (73)	−109	177	0⋅28 (0⋅14–0⋅41)	0⋅30 (0⋅11–0⋅50)
Vegetables (g)	76 %	7 %	313 (278)	−231	857	0⋅16 (0⋅09–0⋅23)	0⋅22 (0⋅11–0⋅33)
Fruits and berries (g)	91 %	0 %	158 (209)	−251	567	0⋅43 (0⋅29–0⋅56)	0⋅58 (0⋅37–0⋅79)
Juice (g)	n/a	n/a	30 (98)	−163	222	0⋅53 (0⋅39–0⋅68)	0⋅67 (0⋅49–0⋅85)
Meat, blood, offal (g)	73 %	6 %	44 (80)	−112	201	0⋅26 (0⋅11–0⋅42)	0⋅54 (0⋅28–0⋅81)
Red meat (g)	72 %	1 %	30 (58)	−84	143	0⋅38 (0⋅22–0⋅55)	0⋅70 (0⋅49–0⋅92)
Fish and shellfish (g)	76 %	7 %	59 (77)	−92	210	0⋅34 (0⋅13–0⋅55)	0⋅62 (0⋅27–0⋅97)
Lean fish (g)	n/a	n/a	27 (47)	−65	119	0⋅14 (−0⋅06 to 0⋅34)	0⋅26 (−0⋅05 to 0⋅58)
Fatty fish (g)	n/a	n/a	16 (31)	−44	76	0⋅50 (0⋅16–0⋅83)	1⋅02 (0⋅56–1⋅48)
Eggs (g)	n/a	n/a	8 (36)	−62	78	0⋅19 (0⋅02–0⋅36)	0⋅54 (0⋅16–0⋅92)
Milk, yoghurt (g)	87 %	1 %	150 (334)	−504	804	0⋅25 (0⋅14–0⋅36)	0⋅43 (0⋅28–0⋅58)
Cheese (g)	85 %	1 %	−10 (29)	−66	47	0⋅61 (0⋅34–0⋅87)	0⋅90 (0⋅52–1⋅27)
Butter, margarine, oil (g)	87 %	1 %	29 (42)	−54	112	0⋅15 (0⋅07–0⋅23)	0⋅37 (0⋅23–0⋅51)

FFQ, food frequency questionnaire; 24-HDR, 24-hour dietary recall; sd, standard deviation; 95 % CI, 95 % confidence interval; LOA, limits of agreement; MUFA, monounsaturated fatty acids; PUFA, polyunsaturated fatty acids.

a Energy-adjusted calibration coefficient.

n/a, not available as cross-classification could not be computed for variables because of non-reporting in the 24-HDRs.

† WebFFQ – 24-HDR.

‡ Mean difference ± 1⋅96 sd of the difference.

Linear calibration coefficients ranged from 0⋅10 (wholegrain) to 0⋅81 (alcohol) for nutrients, and from 0⋅10 (bread) to 0⋅61 (cheese) for foods (Table 3). When energy-adjusted, the calibration coefficients increased for all nutrients and foods. All calibration coefficients were below 1, except for the energy-adjusted value for ‘fatty fish’.

**Fig. 1.**
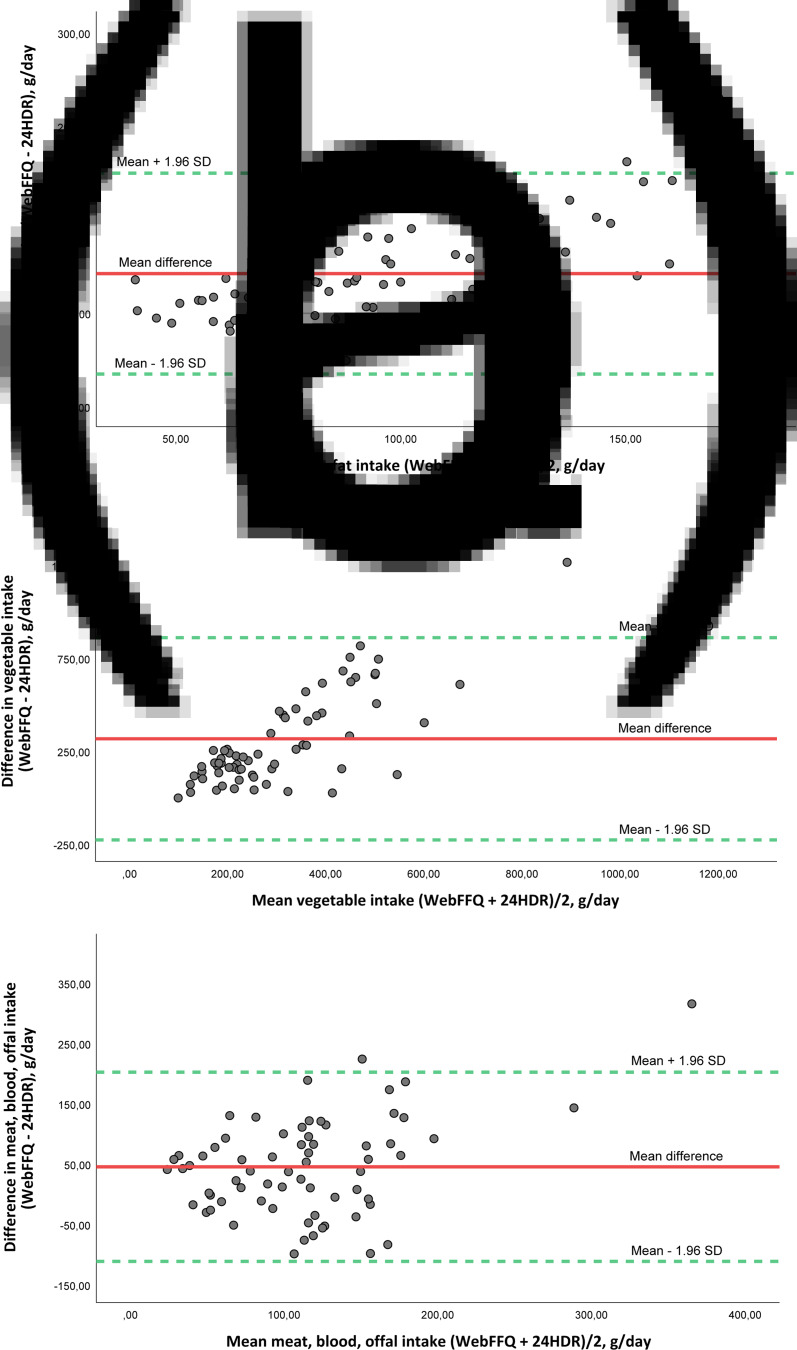
(a) Bland–Altman plot of agreement between fat intake estimated from the web-based food frequency questionnaire (WebFFQ) and the three 24-hour dietary recall interviews (24-HDRs) (*n* 67) in the Hordaland Health Study 3. (b) Bland–Altman plot of agreement between vegetable intake estimated from the web-based food frequency questionnaire (WebFFQ) and the three 24-hour dietary recall interviews (24-HDRs) (*n* 67) in the Hordaland Health Study 3. (c) Bland–Altman plot of agreement between meat, blood, offal intake estimated from the web-based food frequency questionnaire (WebFFQ) and the three 24-hour dietary recall interviews (24-HDRs) (*n* 67) in the Hordaland Health Study 3. The solid line shows the mean difference between the two methods, and the dotted lines show limits of agreement (LOA) corresponding to ±1⋅96 sd.

LOAs were wide, and visual inspection of Bland–Altman plots indicated various patterns: (i) no relationship between differences and low and moderate mean intake, but increasing positive differences with increasing mean intake (e.g. fat and bread) (Fig. 1(a)), indicating greater overestimation by the WebFFQ compared with the 24-HDRs for high intake quantities, (ii) increasing positive differences with increasing mean intake values (e.g. vegetables and protein) (Fig. 1(b)), indicating that the overestimations by the WebFFQ increase consistently with increasing intake quantity and (iii) increased spacing of scatter with increasing intakes, implying that the agreement decreases with increasing intake quantities (e.g. meat and added sugar) (Fig. 1(c)). Few participants were found to lie beyond LOAs.

## Discussion

The results of the present comparison study suggest that the WebFFQ administered in HUSK3 performed reasonably well in estimating and ranking intakes of a variety of nutrients and foods when compared with estimated intakes from three repeated 24-HDRs.

The calculated intakes indicated that the WebFFQ significantly overestimated the absolute intakes of all nutrients and most foods, while it underestimated the intake of a few foods. Similar observations of overestimation by FFQs compared with other dietary assessment methods have been made in other cohort studies^(3,29–33)^, although some studies have reported underestimations by FFQs^(34,35)^. Our finding of general overestimation of nutrients and foods by the WebFFQ compared with repeated 24-HDRs is particularly consistent with the findings of Medin *et al.*^(3)^, who assessed the validity of the same WebFFQ in a different population using both doubly labelled water (DLW) and multiple 24-HDRs as reference methods.

The predominant overestimation of the absolute intake estimated by the WebFFQ in our study may be at least partly explained by the large number of food items listed and the inclusion of rarely consumed food items in the WebFFQ, which may not have been captured by the three repetitions of the 24-HDR^(6,36)^. Furthermore, we observed that amount of intake seemed to be related to the number of items in the WebFFQ, with higher intakes generally being evident for food groups with a large number of listed items. Medin *et al.*^(3)^ observed that 24-HDR underestimated energy intake compared with DLW in their study, reminding us that the reference method, the 24-HDR, is also affected by sources of error^(15)^. This may indicate that the difference in dietary intake estimates between the WebFFQ and the 24-HDRs in our study may have been further increased due to underestimation by the 24-HDRs. The differences in absolute intake estimates decreased when energy adjusted. As described by Willett *et al.*^(27)^, the intake of several nutrients, in particular energy-providing nutrients, are correlated with total energy intake. Although energy adjustment has mainly been discussed in the context of energy-providing nutrients, many dietary components tend to be positively correlated with total energy intake: Individuals who have/report a higher intake of foods generally will have a higher total energy intake – consequently leading to a higher intake of many nutrients^(27)^.

Additionally, the WebFFQ and 24-HDRs may have been disproportionately affected by social desirability bias. Considering that the WebFFQ was self-administered, whereas the 24-HDRs were interviewer-administered and conducted by a clinical dietitian, participants may have been more prone to underreporting their intake of energy-dense foods during the 24-HDRs^(15,37)^.

Estimated correlation coefficients between the WebFFQ and 24-HDRs for absolute intake data ranged from 0⋅19 (iodine) to 0⋅69 (vitamin D) for nutrients and from 0⋅31 (fatty fish) to 0⋅71 (juice) for foods, with all correlations being categorised as acceptable or strong, apart from ‘iodine’. This is consistent with the findings of the study by Medin *et al.*^(3)^ in which correlation coefficients ranging from 0⋅19 (fibre) to 0⋅69 (juice) were reported. Similar to the findings of Medin *et al.*, the highest correlation coefficient in our study was observed for ‘juice’^(3)^. A recent systematic review and meta-analysis reported FFQ validity correlation coefficients ranging from 0⋅22 to 0⋅77 for the sixty-six included studies that used 24-HDR as a reference method^(38)^. Hence, the correlation coefficients in our study appear to be in accordance with those of other relative validation studies^(39–41)^. Although better correlation estimates have been reported in some studies, such discrepancies may be attributed to methodological differences, such as differences in the length of the FFQ, the number of 24-HDRs used as the reference method, sample size, and the nutrients and foods included in the validity assessment.

The weaker ranking ability of our WebFFQ for iodine may be partly explained by differences in the time frame covered by the two methods. Along with milk and dairy products, lean fish are among the most important sources of iodine in the Norwegian diet^(42,43)^. Adequate concentrations of iodine only occur naturally in a selection of foods, with the highest concentrations being found in marine fish^(44)^. While the WebFFQ covered dietary intake in the long run, the 24-HDR covered short-term intake and may not have been able to capture episodically consumed foods such as lean fish. Hence, the relative validity of FFQs is generally better for frequently ingested nutrients and foods.

While correlation coefficients may reflect the agreement in ranking between the WebFFQ and the repeated 24-HDRs^(8)^, the Bland–Altman method is preferred when evaluating the degree to which the two methods agree across a range of intakes^(6)^. Mean differences were positive for all nutrients and foods except alcohol and ‘cheese’, corroborating the finding that the WebFFQ generally yielded a higher intake than the 24-HDRs. Although several patterns were evident in the plots, we generally observed that the mean differences between the WebFFQ and 24-HDRs increased with increasing intake quantities. Other studies have reported comparable findings, suggesting an increasing bias with increasing mean intake^(30,41,45)^.

As mentioned above, the validity of the WebFFQ has been assessed in a previous study, although in a different age group^(3)^. The background of the study participants may naturally influence the quality of response to dietary questionnaires which may in turn influence the magnitude of systematic and random errors^(46)^. In addition, food preferences and availability may differ considerably between settings^(29)^. Furthermore, completion of the WebFFQ administered in HUSK3 required a certain degree of digital literacy, which may be indicative of a high educational level and socioeconomic status among our study participants^(47)^. Hence, validity estimates from one population may generally not be extrapolated to other populations, and it has, therefore, been recommended that questionnaires be validated in subsamples that are representative of the main study^(48)^. Such representativeness was ensured in the present study by including participants from the original HUSK3 cohort, in which the WebFFQ was administered. Representativeness was further corroborated by the similarity in BMI, waist circumference and smoking status between our study subgroup and the total HUSK3 cohort. Although the validity of the WebFFQ has previously been assessed by Medin *et al.*^(3)^, there are considerable differences between the two study populations, especially with regard to age. While the study by Medin *et al.* includes subjects between the ages of 18–70 years, HUSK3 operates with a narrow age group (67–70 years). An added advantage of conducting the comparison study in a subsample of the main study population in which risk analyses will be performed, is the opportunity to perform regression calibration to account for measurement errors in the dietary data^(49,50)^.

Another key strength of our study is the use of a WebFFQ specifically tailored to suit the Norwegian food culture. The use of images in the WebFFQ and picture booklets for the telephonic 24-HDRs may have led to a more accurate portion size estimation and reduced burden on participants. However, the use of the picture booklet was limited to the telephonic 24-HDRs. Using the picture booklet in the first in-person 24-HDR as well, may have contributed to further standardisation and improvements in portion size estimation by the reference method. The moderate agreement between the WebFFQ and 24-HDRs in our study may be attributed to day-to-day variations in dietary intake, which potentially means that three repetitions of the 24-HDR method might not have provided an adequate impression of habitual diet in the same manner as the WebFFQ. Although it has been proposed that increasing the number of recalls may enhance agreement between methods^(51)^, it may also lead to lower response quality and increase the probability of withdrawal due to the excessive burden on participants. Inclusion of all participants who completed at least two 24-HDRs in the analysis led to a subtle decline in correlation coefficients for most of the nutrients and foods (data not shown), which may indicate the importance of conducting at least three repetitions of the 24-HDR. A key limitation in our study is the lack of biomarkers to examine the validity. Although the use of biomarkers may have provided added knowledge about the validity of the WebFFQ, it was deemed unfeasible due to the scarcity of biomarkers reflecting overall dietary intake, but also due to expenses and respondent burden^(9,52)^.

The current comparison study suggests that the WebFFQ administered in HUSK3 is a valuable tool for estimating habitual intake of most of the included nutrients and foods, whereas weaker ranking abilities were observed for others, e.g. iodine. Nutritional epidemiology typically aims to assess the association between different intake levels and health outcomes, implying that the acceptable ranking of individuals according to intake levels is more important than the absolute dietary intake^(39)^. Dietary data collected in HUSK3 are mainly intended for use in analyses of diet and health-related outcomes. Hence, the present study fills an important knowledge gap in the assessment of dietary intake within this cohort and may provide a better understanding of diet-disease associations investigated in future studies using data from HUSK3.
